# (*S*)-Benzyl 3-phenyl­carbamoyl-1,2,3,4-tetra­hydro­isoquinoline-2-carboxyl­ate

**DOI:** 10.1107/S1600536812007969

**Published:** 2012-02-29

**Authors:** Tricia Naicker, Madichaba Chelopo, Thavendran Govender, Hendrick G. Kruger, Glenn E. M. Maguire

**Affiliations:** a School of Pharmacy and Pharmacology, University of KwaZulu-Natal, Durban 4000, South Africa; b School of Chemistry, University of KwaZulu-Natal, Durban 4000, South Africa

## Abstract

There are two independent mol­ecules in the asymmetric unit of the title compound, C_24_H_22_N_2_O_3_. The heterocyclic ring assumes a twisted boat conformation and N—H⋯O inter­actions help to construct the three-dimensional network within the crystal packing.

## Related literature

For background literature, see: Sridharan *et al.* (2011 ▶). For related literature on the synthesis, see: Peters *et al.* (2010 ▶). For related crystal strucutures, see: Naicker *et al.* (2011*a* ▶, 2011*b* ▶).
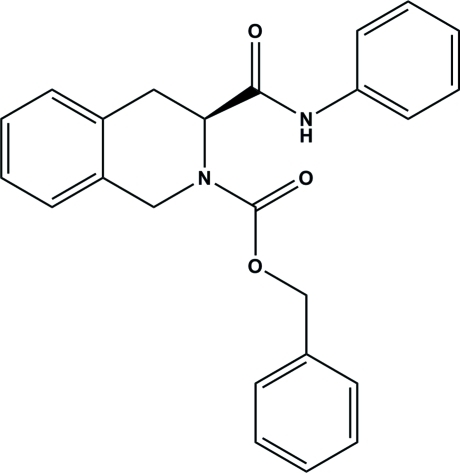


## Experimental

### 

#### Crystal data

C_24_H_22_N_2_O_3_
*M* *_r_* = 386.44
Monoclinic, 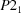

*a* = 13.0338 (5) Å
*b* = 10.8079 (2) Å
*c* = 14.0431 (5) Å
β = 92.031 (1)°
*V* = 1976.98 (11) Å^3^
*Z* = 4
Mo *K*α radiation
μ = 0.09 mm^−1^
*T* = 173 K
0.34 × 0.34 × 0.23 mm

#### Data collection

Nonius KappaCCD diffractometer
9418 measured reflections
4958 independent reflections
4347 reflections with *I* > 2σ(*I*)
*R*_int_ = 0.014

#### Refinement

*R*[*F* ^2^ > 2σ(*F* ^2^)] = 0.039
*wR*(*F* ^2^) = 0.095
*S* = 1.15
4958 reflections
532 parameters
3 restraints
H atoms treated by a mixture of independent and constrained refinement
Δρ_max_ = 0.54 e Å^−3^
Δρ_min_ = −0.57 e Å^−3^

### 

Data collection: *COLLECT* (Nonius, 2000 ▶); cell refinement: *DENZO–SMN* (Otwinowski & Minor, 1997 ▶); data reduction: *DENZO–SMN*; program(s) used to solve structure: *SHELXS97* (Sheldrick, 2008 ▶); program(s) used to refine structure: *SHELXL97* (Sheldrick, 2008 ▶); molecular graphics: *OLEX2* (Dolomanov *et al.*, 2009 ▶); software used to prepare material for publication: *SHELXL97*.

## Supplementary Material

Crystal structure: contains datablock(s) I, global. DOI: 10.1107/S1600536812007969/hg5180sup1.cif

Structure factors: contains datablock(s) I. DOI: 10.1107/S1600536812007969/hg5180Isup2.hkl

Supplementary material file. DOI: 10.1107/S1600536812007969/hg5180Isup3.cml

Additional supplementary materials:  crystallographic information; 3D view; checkCIF report

## Figures and Tables

**Table 1:** Hydrogen-bond geometry (Å, °)

*D*—H⋯*A*	*D*—H	H⋯*A*	*D*⋯*A*	*D*—H⋯*A*
N2*A*—H2*A*⋯O1*B*^i^	0.97 (1)	1.97 (1)	2.9213 (16)	167 (1)
N2*B*—H2*B*⋯O2*B*^ii^	0.97 (1)	1.92 (1)	2.8904 (16)	176 (1)

Symmetry codes: (i) 
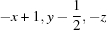
; (ii) 
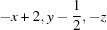
.

**Fig. 1.**
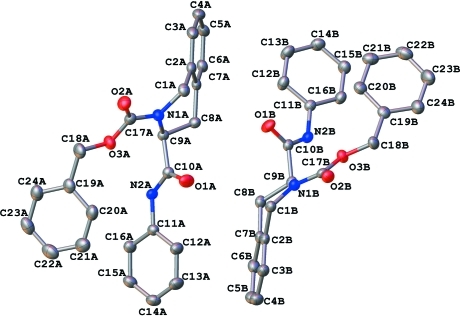
The molecular structure of the title compound with the atom numbering scheme. Displacement ellipsoids are drawn at the 40% probability level. Hydrogen atoms have been omitted for clarity.

**Fig. 2.**
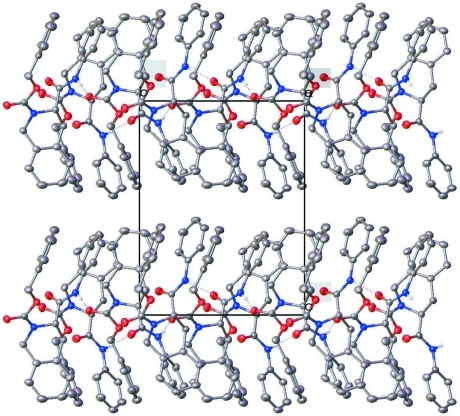
A partial projection of the title compound in the [100] plane.

## supplementary crystallographic information

### Comment

The tetrahydroisoquinoline (TIQ) molecule and its derivatives have been widely investigated for their biological and pharmaceutical properties (Sridharan *et al.* 2011). Our research currently focuses on the evaluation of novel TIQ compounds for their potential biological activity and as a source of chirality in the synthesis of novel asymmetric catalysts.

The title compound is a precursor in the synthesis of novel asymmetric catalysts and containing a tetrahydroisoquinoline framework (Peters *et al.* 2010).

The title structure was derived from commercially available *S*-phenyl glycine and formaldehyde. The absolute stereochemistry was confirmed to be S at the C9 position from proton NMR spectroscopy (Peters *et al.* 2010).

The structure has two molecules in the asymmetric unit (Fig. 1). The molecules display intermolecular hydrogen bonding *via* the amide and carbamate carbonyl group. (Table 1). This bonding arrangement creates chains which link the molecules together resulting in layers parallel to the 100 plane. (Fig. 2).

From the crystal structure it is evident that the *N*-containing six membered ring assumes a twisted boat conformation [(Q) = 0.6507 (15) Å, θ = 90.29 (13)°, φ = 237.65 (13)°] which is peculiar since this ring in similar TIQ deriviatives displays either a half chair or boat form (Naicker *et al.* 2011*a* and 2011*b*). The *N*-Cbz group is staggered to the ester group at the C9 position, the torsion angle is H1A—C1A—N1A—C17A -67.5 (2)°.

### Experimental

(*S*)-2-(Benzyloxycarbonyl)-1,2,3,4-tetrahydroisoquinoline-3- carboxylic acid (1.5 g, 4.8 mmol) was dissolved in DMF (15 ml) followed by addition of EDC.HCl (1.1 g, 5.8 mmol), HOBt (0.81 g, 5.3 mmol), a catalytic amount of DMAP and aniline (5.3 mmol). The reaction mixture was then stirred at room temperature until no more starting material could be detected by TLC analysis (approximately 1 h). The reaction mixture was poured into 30 volumes of chilled water; the mixture was then extracted twice with ethyl acetate. The extracts were combined, washed with 10% aqueous HCl to remove latent EDC urea, dried over anhydrous magnesium sulfate and then concentrated to dryness affording the crude product which was purified by column chromatography. (Hexane:EtOAc 60:40 *R*_f_ 1/2).

Melting point = 410–412 K

Recrystallization from ethyl acetate at room temperature afforded crystals suitable for X-ray analysis.

### Refinement

All non-hydrogen atoms were refined anisotropically. The hydrogen atoms H2A and H2B were located in the difference density maps and refined with simple bond length constraints with d(N—H) = 0.970 (2) Å. The remaining hydrogen atoms could all be found in the difference electron density maps but were finally placed in idealized positions and refined in riding models with *U*_iso_ set at 1.2 or 1.5 times those of their parent atoms. The Friedel pairs were merged.

### Crystal data

**Table:**

C_24_H_22_N_2_O_3_	*F*(000) = 816
*M**_r_* = 386.44	*D*_x_ = 1.298 Mg m^−^^3^
Monoclinic, *P*2_1_	Melting point: 411 K
Hall symbol: P 2yb	Mo *K*α radiation, λ = 0.71073 Å
*a* = 13.0338 (5) Å	Cell parameters from 9418 reflections
*b* = 10.8079 (2) Å	θ = 2.5–27.9°
*c* = 14.0431 (5) Å	µ = 0.09 mm^−^^1^
β = 92.031 (1)°	*T* = 173 K
*V* = 1976.98 (11) Å^3^	Block, colourless
*Z* = 4	0.34 × 0.34 × 0.23 mm

### Data collection

**Table:**

Nonius KappaCCD diffractometer	4347 reflections with *I* > 2σ(*I*)
Radiation source: fine-focus sealed tube	*R*_int_ = 0.014
Graphite monochromator	θ_max_ = 27.9°, θ_min_ = 2.5°
1.2° φ scans and ω scans	*h* = −17→17
9418 measured reflections	*k* = −14→14
4958 independent reflections	*l* = −18→18

### Refinement

**Table:**

Refinement on *F*^2^	Secondary atom site location: difference Fourier map
Least-squares matrix: full	Hydrogen site location: inferred from neighbouring sites
*R*[*F*^2^ > 2σ(*F*^2^)] = 0.039	H atoms treated by a mixture of independent and constrained refinement
*wR*(*F*^2^) = 0.095	*w* = 1/[σ^2^(*F*_o_^2^) + (0.0622*P*)^2^] where *P* = (*F*_o_^2^ + 2*F*_c_^2^)/3
*S* = 1.15	(Δ/σ)_max_ = 0.001
4958 reflections	Δρ_max_ = 0.54 e Å^−^^3^
532 parameters	Δρ_min_ = −0.57 e Å^−^^3^
3 restraints	Extinction correction: *SHELXL97* (Sheldrick, 2008), Fc^*^=kFc[1+0.001xFc^2^λ^3^/sin(2θ)]^-1/4^
Primary atom site location: structure-invariant direct methods	Extinction coefficient: 0.046 (3)

### Special details

**Table:**

Geometry. All e.s.d.'s (except the e.s.d. in the dihedral angle between two l.s. planes) are estimated using the full covariance matrix. The cell e.s.d.'s are taken into account individually in the estimation of e.s.d.'s in distances, angles and torsion angles; correlations between e.s.d.'s in cell parameters are only used when they are defined by crystal symmetry. An approximate (isotropic) treatment of cell e.s.d.'s is used for estimating e.s.d.'s involving l.s. planes.
Refinement. Refinement of *F*^2^ against ALL reflections. The weighted *R*-factor *wR* and goodness of fit *S* are based on *F*^2^, conventional *R*-factors *R* are based on *F*, with *F* set to zero for negative *F*^2^. The threshold expression of *F*^2^ > σ(*F*^2^) is used only for calculating *R*-factors(gt) *etc*. and is not relevant to the choice of reflections for refinement. *R*-factors based on *F*^2^ are statistically about twice as large as those based on *F*, and *R*- factors based on ALL data will be even larger.

### Fractional atomic coordinates and isotropic or equivalent isotropic displacement parameters (Å^2^)

**Table:**

	*x*	*y*	*z*	*U*_iso_*/*U*_eq_	
O1A	0.52998 (11)	0.38204 (15)	0.11077 (10)	0.0406 (4)	
O2A	0.29082 (11)	0.58944 (14)	−0.03510 (10)	0.0403 (4)	
O3A	0.27453 (11)	0.43269 (13)	0.07094 (10)	0.0361 (3)	
N1A	0.38659 (12)	0.41285 (15)	−0.04295 (11)	0.0293 (3)	
N2A	0.43006 (11)	0.22263 (14)	0.16104 (10)	0.0271 (3)	
H2A	0.3726 (11)	0.1696 (17)	0.1431 (14)	0.036 (6)*	
C1A	0.43931 (15)	0.45372 (19)	−0.12855 (14)	0.0327 (4)	
H1A1	0.5124	0.4710	−0.1122	0.039*	
H1A2	0.4073	0.5307	−0.1537	0.039*	
C2A	0.43120 (14)	0.35392 (19)	−0.20221 (13)	0.0303 (4)	
C3A	0.40204 (16)	0.3749 (2)	−0.29716 (14)	0.0376 (5)	
H3A	0.3880	0.4566	−0.3189	0.045*	
C4A	0.39357 (17)	0.2763 (2)	−0.35980 (15)	0.0419 (5)	
H4A	0.3740	0.2907	−0.4247	0.050*	
C5A	0.41327 (16)	0.1573 (2)	−0.32889 (14)	0.0386 (5)	
H5A	0.4069	0.0901	−0.3723	0.046*	
C6A	0.44253 (14)	0.1354 (2)	−0.23377 (13)	0.0320 (4)	
H6A	0.4559	0.0535	−0.2122	0.038*	
C7A	0.45189 (13)	0.23411 (19)	−0.17097 (12)	0.0283 (4)	
C8A	0.48319 (14)	0.21942 (18)	−0.06702 (12)	0.0292 (4)	
H8A1	0.5541	0.2504	−0.0562	0.035*	
H8A2	0.4824	0.1306	−0.0499	0.035*	
C9A	0.41022 (14)	0.29101 (18)	−0.00241 (12)	0.0265 (4)	
H9A	0.3451	0.2431	0.0034	0.032*	
C10A	0.46280 (14)	0.30510 (17)	0.09645 (12)	0.0275 (4)	
C11A	0.46730 (14)	0.20560 (18)	0.25604 (12)	0.0285 (4)	
C12A	0.55769 (15)	0.2588 (2)	0.29217 (14)	0.0376 (5)	
H12A	0.5975	0.3108	0.2533	0.045*	
C13A	0.58892 (17)	0.2347 (2)	0.38603 (15)	0.0444 (5)	
H13A	0.6505	0.2713	0.4110	0.053*	
C14A	0.53305 (17)	0.1593 (2)	0.44374 (14)	0.0431 (5)	
H14A	0.5557	0.1437	0.5076	0.052*	
C15A	0.44321 (17)	0.1067 (2)	0.40710 (14)	0.0418 (5)	
H15A	0.4040	0.0541	0.4460	0.050*	
C16A	0.41032 (15)	0.1303 (2)	0.31416 (13)	0.0351 (4)	
H16A	0.3481	0.0945	0.2899	0.042*	
C17A	0.31546 (14)	0.48742 (19)	−0.00535 (13)	0.0303 (4)	
C18A	0.20156 (17)	0.5056 (2)	0.12288 (15)	0.0445 (5)	
H18A	0.1320	0.4979	0.0928	0.053*	
H18B	0.2215	0.5940	0.1227	0.053*	
C19A	0.20244 (16)	0.4573 (2)	0.22276 (15)	0.0376 (5)	
C20A	0.29385 (17)	0.4508 (2)	0.27638 (16)	0.0448 (5)	
H20A	0.3562	0.4757	0.2490	0.054*	
C21A	0.2948 (2)	0.4084 (3)	0.36910 (17)	0.0517 (6)	
H21A	0.3577	0.4044	0.4052	0.062*	
C22A	0.2051 (2)	0.3718 (3)	0.40947 (17)	0.0554 (6)	
H22A	0.2060	0.3425	0.4733	0.066*	
C23A	0.1148 (2)	0.3778 (3)	0.35762 (17)	0.0528 (6)	
H23A	0.0528	0.3524	0.3854	0.063*	
C24A	0.11304 (18)	0.4206 (2)	0.26446 (17)	0.0448 (5)	
H24A	0.0497	0.4248	0.2290	0.054*	
O1B	0.72563 (11)	0.54812 (14)	−0.09056 (10)	0.0384 (3)	
O2B	0.96842 (10)	0.80430 (13)	0.02030 (9)	0.0331 (3)	
O3B	0.97586 (10)	0.62280 (13)	−0.06036 (9)	0.0312 (3)	
N1B	0.86643 (11)	0.64064 (15)	0.05632 (10)	0.0278 (3)	
N2B	0.83736 (12)	0.38701 (15)	−0.10304 (11)	0.0280 (3)	
H2B	0.9045 (7)	0.360 (2)	−0.0796 (14)	0.035 (6)*	
C1B	0.82427 (14)	0.70473 (19)	0.13913 (12)	0.0292 (4)	
H1B1	0.7513	0.7258	0.1253	0.035*	
H1B2	0.8624	0.7827	0.1511	0.035*	
C2B	0.83260 (13)	0.6241 (2)	0.22607 (13)	0.0307 (4)	
C3B	0.86246 (15)	0.6692 (2)	0.31573 (13)	0.0378 (5)	
H3B	0.8809	0.7538	0.3235	0.045*	
C4B	0.86516 (17)	0.5907 (3)	0.39320 (15)	0.0465 (6)	
H4B	0.8843	0.6216	0.4546	0.056*	
C5B	0.84012 (17)	0.4673 (3)	0.38180 (16)	0.0471 (6)	
H5B	0.8427	0.4136	0.4354	0.057*	
C6B	0.81103 (16)	0.4211 (2)	0.29225 (15)	0.0404 (5)	
H6B	0.7948	0.3359	0.2845	0.048*	
C7B	0.80592 (13)	0.5004 (2)	0.21426 (13)	0.0315 (4)	
C8B	0.77060 (14)	0.46164 (19)	0.11544 (13)	0.0308 (4)	
H8B1	0.7011	0.4953	0.1011	0.037*	
H8B2	0.7664	0.3703	0.1123	0.037*	
C9B	0.84478 (13)	0.50845 (17)	0.04082 (12)	0.0264 (4)	
H9B	0.9105	0.4611	0.0476	0.032*	
C10B	0.79667 (13)	0.48517 (18)	−0.05885 (13)	0.0268 (4)	
C11B	0.80969 (14)	0.33996 (17)	−0.19506 (13)	0.0272 (4)	
C12B	0.71076 (15)	0.3490 (2)	−0.23410 (15)	0.0362 (4)	
H12B	0.6583	0.3880	−0.1997	0.043*	
C13B	0.68944 (16)	0.3003 (2)	−0.32388 (16)	0.0437 (5)	
H13B	0.6219	0.3073	−0.3511	0.052*	
C14B	0.76409 (16)	0.2419 (2)	−0.37446 (14)	0.0387 (5)	
H14B	0.7484	0.2095	−0.4362	0.046*	
C15B	0.86218 (15)	0.2309 (2)	−0.33438 (14)	0.0374 (5)	
H15B	0.9141	0.1899	−0.3682	0.045*	
C16B	0.88465 (15)	0.27978 (19)	−0.24505 (14)	0.0332 (4)	
H16B	0.9521	0.2720	−0.2177	0.040*	
C17B	0.93931 (13)	0.69790 (18)	0.00696 (12)	0.0258 (4)	
C18B	1.05654 (15)	0.6733 (2)	−0.11746 (13)	0.0341 (4)	
H18C	1.1244	0.6583	−0.0856	0.041*	
H18D	1.0472	0.7637	−0.1251	0.041*	
C19B	1.05156 (15)	0.61123 (18)	−0.21351 (13)	0.0326 (4)	
C20B	0.95807 (17)	0.5933 (2)	−0.26206 (14)	0.0387 (5)	
H20B	0.8960	0.6143	−0.2325	0.046*	
C21B	0.95489 (19)	0.5449 (2)	−0.35342 (15)	0.0463 (5)	
H21B	0.8906	0.5319	−0.3859	0.056*	
C22B	1.0445 (2)	0.5156 (3)	−0.39740 (16)	0.0524 (6)	
H22B	1.0422	0.4838	−0.4605	0.063*	
C23B	1.1380 (2)	0.5328 (3)	−0.34923 (18)	0.0543 (6)	
H23B	1.1999	0.5130	−0.3794	0.065*	
C24B	1.14128 (18)	0.5788 (2)	−0.25690 (16)	0.0442 (5)	
H24B	1.2054	0.5881	−0.2234	0.053*	

### Atomic displacement parameters (Å^2^)

**Table:**

	*U*^11^	*U*^22^	*U*^33^	*U*^12^	*U*^13^	*U*^23^
O1A	0.0425 (8)	0.0410 (8)	0.0379 (8)	−0.0185 (7)	−0.0053 (6)	0.0001 (7)
O2A	0.0396 (8)	0.0343 (8)	0.0468 (8)	0.0089 (6)	0.0009 (6)	0.0023 (7)
O3A	0.0377 (8)	0.0349 (7)	0.0362 (7)	0.0066 (6)	0.0100 (6)	0.0001 (6)
N1A	0.0309 (8)	0.0277 (8)	0.0296 (8)	0.0016 (6)	0.0033 (6)	0.0015 (6)
N2A	0.0261 (7)	0.0283 (8)	0.0267 (7)	−0.0031 (6)	−0.0026 (6)	−0.0015 (6)
C1A	0.0318 (9)	0.0300 (10)	0.0369 (10)	−0.0014 (8)	0.0071 (8)	0.0061 (8)
C2A	0.0227 (9)	0.0369 (10)	0.0317 (9)	−0.0011 (8)	0.0053 (7)	0.0047 (8)
C3A	0.0330 (10)	0.0470 (12)	0.0330 (10)	0.0055 (9)	0.0055 (8)	0.0112 (9)
C4A	0.0368 (11)	0.0620 (15)	0.0269 (10)	0.0019 (10)	0.0009 (8)	0.0073 (9)
C5A	0.0359 (11)	0.0520 (13)	0.0281 (9)	−0.0042 (10)	0.0040 (8)	−0.0061 (9)
C6A	0.0292 (9)	0.0362 (10)	0.0310 (9)	−0.0017 (8)	0.0069 (7)	−0.0010 (8)
C7A	0.0219 (8)	0.0354 (10)	0.0278 (9)	0.0001 (8)	0.0050 (7)	0.0018 (8)
C8A	0.0290 (9)	0.0307 (10)	0.0276 (9)	0.0032 (8)	−0.0014 (7)	0.0005 (8)
C9A	0.0272 (9)	0.0263 (9)	0.0258 (8)	−0.0006 (7)	−0.0003 (7)	−0.0005 (7)
C10A	0.0276 (9)	0.0260 (9)	0.0290 (9)	−0.0007 (7)	0.0003 (7)	−0.0033 (7)
C11A	0.0297 (9)	0.0299 (9)	0.0259 (8)	0.0028 (8)	0.0007 (7)	−0.0029 (7)
C12A	0.0331 (10)	0.0456 (12)	0.0339 (10)	−0.0074 (9)	−0.0034 (8)	0.0026 (9)
C13A	0.0401 (11)	0.0568 (14)	0.0354 (11)	−0.0064 (11)	−0.0091 (9)	−0.0029 (10)
C14A	0.0485 (13)	0.0555 (14)	0.0251 (9)	0.0025 (11)	−0.0023 (8)	−0.0030 (9)
C15A	0.0470 (12)	0.0497 (13)	0.0292 (9)	−0.0031 (11)	0.0065 (8)	0.0003 (9)
C16A	0.0339 (10)	0.0395 (11)	0.0318 (9)	−0.0058 (9)	0.0005 (7)	−0.0024 (8)
C17A	0.0279 (9)	0.0301 (10)	0.0328 (9)	−0.0003 (8)	−0.0024 (7)	−0.0018 (8)
C18A	0.0420 (12)	0.0477 (13)	0.0445 (12)	0.0145 (10)	0.0128 (9)	−0.0019 (11)
C19A	0.0381 (11)	0.0344 (11)	0.0408 (11)	0.0050 (9)	0.0088 (8)	−0.0091 (9)
C20A	0.0340 (11)	0.0525 (14)	0.0483 (12)	0.0062 (10)	0.0069 (9)	−0.0081 (10)
C21A	0.0509 (14)	0.0598 (15)	0.0444 (12)	0.0135 (12)	0.0005 (10)	−0.0093 (12)
C22A	0.0738 (18)	0.0526 (15)	0.0406 (12)	0.0127 (13)	0.0136 (12)	−0.0051 (11)
C23A	0.0530 (14)	0.0539 (15)	0.0530 (14)	−0.0052 (12)	0.0228 (12)	−0.0081 (12)
C24A	0.0379 (11)	0.0492 (13)	0.0478 (12)	−0.0010 (10)	0.0071 (9)	−0.0114 (11)
O1B	0.0355 (8)	0.0372 (8)	0.0418 (8)	0.0137 (6)	−0.0108 (6)	−0.0076 (6)
O2B	0.0318 (7)	0.0322 (7)	0.0351 (7)	−0.0066 (6)	−0.0020 (5)	−0.0033 (6)
O3B	0.0314 (7)	0.0310 (7)	0.0315 (6)	−0.0041 (6)	0.0074 (5)	−0.0031 (6)
N1B	0.0278 (7)	0.0276 (8)	0.0278 (7)	−0.0016 (6)	0.0013 (6)	−0.0023 (6)
N2B	0.0245 (7)	0.0296 (8)	0.0298 (7)	0.0036 (6)	−0.0023 (6)	−0.0009 (7)
C1B	0.0251 (9)	0.0343 (10)	0.0282 (9)	0.0038 (8)	0.0002 (7)	−0.0041 (8)
C2B	0.0208 (8)	0.0412 (11)	0.0304 (9)	0.0020 (8)	0.0020 (7)	−0.0014 (8)
C3B	0.0292 (10)	0.0521 (13)	0.0319 (10)	−0.0004 (9)	−0.0005 (8)	−0.0035 (9)
C4B	0.0402 (12)	0.0689 (16)	0.0303 (10)	−0.0015 (11)	−0.0028 (9)	−0.0006 (11)
C5B	0.0406 (12)	0.0653 (16)	0.0355 (11)	0.0028 (11)	0.0018 (9)	0.0143 (11)
C6B	0.0341 (11)	0.0471 (13)	0.0405 (11)	0.0006 (10)	0.0076 (9)	0.0088 (10)
C7B	0.0207 (8)	0.0429 (11)	0.0312 (9)	0.0014 (8)	0.0036 (7)	0.0012 (8)
C8B	0.0259 (9)	0.0327 (10)	0.0340 (9)	−0.0033 (8)	0.0025 (7)	0.0003 (8)
C9B	0.0235 (8)	0.0255 (9)	0.0301 (9)	0.0010 (7)	0.0004 (7)	0.0002 (7)
C10B	0.0221 (8)	0.0275 (9)	0.0308 (9)	−0.0004 (7)	0.0003 (7)	0.0006 (7)
C11B	0.0273 (9)	0.0229 (8)	0.0311 (9)	−0.0004 (7)	−0.0010 (7)	0.0005 (7)
C12B	0.0268 (10)	0.0383 (11)	0.0433 (11)	0.0022 (8)	−0.0023 (8)	−0.0081 (9)
C13B	0.0324 (11)	0.0472 (13)	0.0504 (13)	0.0009 (10)	−0.0117 (9)	−0.0095 (11)
C14B	0.0428 (11)	0.0390 (11)	0.0338 (10)	−0.0017 (9)	−0.0062 (8)	−0.0077 (9)
C15B	0.0358 (10)	0.0391 (12)	0.0373 (10)	0.0026 (9)	0.0028 (8)	−0.0064 (9)
C16B	0.0271 (9)	0.0358 (11)	0.0364 (10)	0.0034 (8)	−0.0022 (8)	−0.0028 (8)
C17B	0.0216 (8)	0.0307 (9)	0.0248 (8)	−0.0003 (7)	−0.0043 (6)	−0.0002 (7)
C18B	0.0282 (9)	0.0408 (11)	0.0336 (9)	−0.0074 (8)	0.0069 (8)	−0.0003 (8)
C19B	0.0376 (10)	0.0288 (9)	0.0316 (9)	−0.0012 (8)	0.0038 (8)	0.0048 (8)
C20B	0.0391 (11)	0.0431 (12)	0.0342 (10)	−0.0024 (9)	0.0051 (8)	0.0045 (9)
C21B	0.0564 (14)	0.0485 (13)	0.0335 (11)	−0.0035 (11)	−0.0051 (10)	0.0023 (10)
C22B	0.0716 (16)	0.0538 (15)	0.0320 (11)	0.0096 (13)	0.0053 (11)	−0.0044 (10)
C23B	0.0538 (14)	0.0630 (16)	0.0468 (13)	0.0187 (13)	0.0120 (11)	−0.0046 (12)
C24B	0.0419 (12)	0.0506 (13)	0.0405 (11)	0.0070 (10)	0.0054 (9)	0.0003 (10)

### Geometric parameters (Å, º)

**Table:**

O1A—C10A	1.219 (2)	O1B—C10B	1.220 (2)
O2A—C17A	1.218 (2)	O2B—C17B	1.223 (2)
O3A—C17A	1.350 (2)	O3B—C17B	1.346 (2)
O3A—C18A	1.452 (2)	O3B—C18B	1.451 (2)
N1A—C17A	1.350 (2)	N1B—C17B	1.346 (2)
N1A—C9A	1.463 (2)	N1B—C9B	1.471 (2)
N1A—C1A	1.473 (2)	N1B—C1B	1.476 (2)
N2A—C10A	1.352 (2)	N2B—C10B	1.347 (2)
N2A—C11A	1.415 (2)	N2B—C11B	1.423 (2)
N2A—H2A	0.969 (2)	N2B—H2B	0.969 (2)
C1A—C2A	1.496 (3)	C1B—C2B	1.501 (3)
C1A—H1A1	0.9900	C1B—H1B1	0.9900
C1A—H1A2	0.9900	C1B—H1B2	0.9900
C2A—C7A	1.390 (3)	C2B—C7B	1.390 (3)
C2A—C3A	1.392 (3)	C2B—C3B	1.393 (3)
C3A—C4A	1.384 (3)	C3B—C4B	1.380 (3)
C3A—H3A	0.9500	C3B—H3B	0.9500
C4A—C5A	1.379 (4)	C4B—C5B	1.381 (4)
C4A—H4A	0.9500	C4B—H4B	0.9500
C5A—C6A	1.396 (3)	C5B—C6B	1.393 (3)
C5A—H5A	0.9500	C5B—H5B	0.9500
C6A—C7A	1.387 (3)	C6B—C7B	1.390 (3)
C6A—H6A	0.9500	C6B—H6B	0.9500
C7A—C8A	1.510 (2)	C7B—C8B	1.506 (3)
C8A—C9A	1.545 (2)	C8B—C9B	1.536 (2)
C8A—H8A1	0.9900	C8B—H8B1	0.9900
C8A—H8A2	0.9900	C8B—H8B2	0.9900
C9A—C10A	1.534 (2)	C9B—C10B	1.534 (2)
C9A—H9A	1.0000	C9B—H9B	1.0000
C11A—C16A	1.387 (3)	C11B—C16B	1.385 (3)
C11A—C12A	1.391 (3)	C11B—C12B	1.386 (3)
C12A—C13A	1.390 (3)	C12B—C13B	1.385 (3)
C12A—H12A	0.9500	C12B—H12B	0.9500
C13A—C14A	1.376 (3)	C13B—C14B	1.378 (3)
C13A—H13A	0.9500	C13B—H13B	0.9500
C14A—C15A	1.385 (3)	C14B—C15B	1.384 (3)
C14A—H14A	0.9500	C14B—H14B	0.9500
C15A—C16A	1.383 (3)	C15B—C16B	1.383 (3)
C15A—H15A	0.9500	C15B—H15B	0.9500
C16A—H16A	0.9500	C16B—H16B	0.9500
C18A—C19A	1.497 (3)	C18B—C19B	1.505 (3)
C18A—H18A	0.9900	C18B—H18C	0.9900
C18A—H18B	0.9900	C18B—H18D	0.9900
C19A—C24A	1.381 (3)	C19B—C24B	1.383 (3)
C19A—C20A	1.388 (3)	C19B—C20B	1.389 (3)
C20A—C21A	1.380 (3)	C20B—C21B	1.385 (3)
C20A—H20A	0.9500	C20B—H20B	0.9500
C21A—C22A	1.375 (4)	C21B—C22B	1.378 (4)
C21A—H21A	0.9500	C21B—H21B	0.9500
C22A—C23A	1.363 (4)	C22B—C23B	1.384 (4)
C22A—H22A	0.9500	C22B—H22B	0.9500
C23A—C24A	1.387 (3)	C23B—C24B	1.388 (3)
C23A—H23A	0.9500	C23B—H23B	0.9500
C24A—H24A	0.9500	C24B—H24B	0.9500
			
C17A—O3A—C18A	116.42 (16)	C17B—O3B—C18B	116.29 (15)
C17A—N1A—C9A	121.54 (15)	C17B—N1B—C9B	120.36 (15)
C17A—N1A—C1A	119.13 (16)	C17B—N1B—C1B	118.62 (15)
C9A—N1A—C1A	119.31 (15)	C9B—N1B—C1B	119.83 (15)
C10A—N2A—C11A	127.77 (15)	C10B—N2B—C11B	127.47 (16)
C10A—N2A—H2A	118.3 (13)	C10B—N2B—H2B	116.6 (13)
C11A—N2A—H2A	113.9 (13)	C11B—N2B—H2B	113.4 (13)
N1A—C1A—C2A	108.94 (15)	N1B—C1B—C2B	110.49 (16)
N1A—C1A—H1A1	109.9	N1B—C1B—H1B1	109.6
C2A—C1A—H1A1	109.9	C2B—C1B—H1B1	109.6
N1A—C1A—H1A2	109.9	N1B—C1B—H1B2	109.6
C2A—C1A—H1A2	109.9	C2B—C1B—H1B2	109.6
H1A1—C1A—H1A2	108.3	H1B1—C1B—H1B2	108.1
C7A—C2A—C3A	119.81 (19)	C7B—C2B—C3B	120.36 (19)
C7A—C2A—C1A	116.44 (16)	C7B—C2B—C1B	116.80 (17)
C3A—C2A—C1A	123.73 (19)	C3B—C2B—C1B	122.81 (19)
C4A—C3A—C2A	119.8 (2)	C4B—C3B—C2B	119.7 (2)
C4A—C3A—H3A	120.1	C4B—C3B—H3B	120.1
C2A—C3A—H3A	120.1	C2B—C3B—H3B	120.1
C5A—C4A—C3A	120.58 (18)	C3B—C4B—C5B	120.2 (2)
C5A—C4A—H4A	119.7	C3B—C4B—H4B	119.9
C3A—C4A—H4A	119.7	C5B—C4B—H4B	119.9
C4A—C5A—C6A	120.0 (2)	C4B—C5B—C6B	120.4 (2)
C4A—C5A—H5A	120.0	C4B—C5B—H5B	119.8
C6A—C5A—H5A	120.0	C6B—C5B—H5B	119.8
C7A—C6A—C5A	119.5 (2)	C7B—C6B—C5B	119.6 (2)
C7A—C6A—H6A	120.2	C7B—C6B—H6B	120.2
C5A—C6A—H6A	120.2	C5B—C6B—H6B	120.2
C6A—C7A—C2A	120.27 (17)	C2B—C7B—C6B	119.61 (19)
C6A—C7A—C8A	123.26 (18)	C2B—C7B—C8B	116.40 (17)
C2A—C7A—C8A	116.46 (17)	C6B—C7B—C8B	124.0 (2)
C7A—C8A—C9A	111.36 (14)	C7B—C8B—C9B	110.96 (15)
C7A—C8A—H8A1	109.4	C7B—C8B—H8B1	109.4
C9A—C8A—H8A1	109.4	C9B—C8B—H8B1	109.4
C7A—C8A—H8A2	109.4	C7B—C8B—H8B2	109.4
C9A—C8A—H8A2	109.4	C9B—C8B—H8B2	109.4
H8A1—C8A—H8A2	108.0	H8B1—C8B—H8B2	108.0
N1A—C9A—C10A	110.12 (15)	N1B—C9B—C10B	111.37 (15)
N1A—C9A—C8A	110.34 (14)	N1B—C9B—C8B	109.93 (15)
C10A—C9A—C8A	108.46 (14)	C10B—C9B—C8B	108.76 (14)
N1A—C9A—H9A	109.3	N1B—C9B—H9B	108.9
C10A—C9A—H9A	109.3	C10B—C9B—H9B	108.9
C8A—C9A—H9A	109.3	C8B—C9B—H9B	108.9
O1A—C10A—N2A	125.53 (16)	O1B—C10B—N2B	125.23 (17)
O1A—C10A—C9A	120.82 (17)	O1B—C10B—C9B	121.45 (17)
N2A—C10A—C9A	113.60 (15)	N2B—C10B—C9B	113.24 (15)
C16A—C11A—C12A	119.50 (17)	C16B—C11B—C12B	119.69 (17)
C16A—C11A—N2A	117.26 (16)	C16B—C11B—N2B	117.85 (16)
C12A—C11A—N2A	123.23 (17)	C12B—C11B—N2B	122.43 (17)
C13A—C12A—C11A	118.97 (19)	C13B—C12B—C11B	119.14 (19)
C13A—C12A—H12A	120.5	C13B—C12B—H12B	120.4
C11A—C12A—H12A	120.5	C11B—C12B—H12B	120.4
C14A—C13A—C12A	121.7 (2)	C14B—C13B—C12B	121.37 (19)
C14A—C13A—H13A	119.1	C14B—C13B—H13B	119.3
C12A—C13A—H13A	119.1	C12B—C13B—H13B	119.3
C13A—C14A—C15A	118.88 (19)	C13B—C14B—C15B	119.25 (19)
C13A—C14A—H14A	120.6	C13B—C14B—H14B	120.4
C15A—C14A—H14A	120.6	C15B—C14B—H14B	120.4
C16A—C15A—C14A	120.3 (2)	C16B—C15B—C14B	119.96 (19)
C16A—C15A—H15A	119.8	C16B—C15B—H15B	120.0
C14A—C15A—H15A	119.8	C14B—C15B—H15B	120.0
C15A—C16A—C11A	120.60 (18)	C15B—C16B—C11B	120.56 (18)
C15A—C16A—H16A	119.7	C15B—C16B—H16B	119.7
C11A—C16A—H16A	119.7	C11B—C16B—H16B	119.7
O2A—C17A—N1A	125.63 (18)	O2B—C17B—O3B	123.98 (17)
O2A—C17A—O3A	124.19 (18)	O2B—C17B—N1B	125.03 (17)
N1A—C17A—O3A	110.18 (16)	O3B—C17B—N1B	110.99 (15)
O3A—C18A—C19A	107.33 (17)	O3B—C18B—C19B	108.59 (15)
O3A—C18A—H18A	110.2	O3B—C18B—H18C	110.0
C19A—C18A—H18A	110.2	C19B—C18B—H18C	110.0
O3A—C18A—H18B	110.2	O3B—C18B—H18D	110.0
C19A—C18A—H18B	110.2	C19B—C18B—H18D	110.0
H18A—C18A—H18B	108.5	H18C—C18B—H18D	108.4
C24A—C19A—C20A	118.5 (2)	C24B—C19B—C20B	119.29 (19)
C24A—C19A—C18A	121.3 (2)	C24B—C19B—C18B	119.83 (18)
C20A—C19A—C18A	120.2 (2)	C20B—C19B—C18B	120.75 (18)
C21A—C20A—C19A	120.5 (2)	C21B—C20B—C19B	120.3 (2)
C21A—C20A—H20A	119.8	C21B—C20B—H20B	119.8
C19A—C20A—H20A	119.8	C19B—C20B—H20B	119.8
C22A—C21A—C20A	120.4 (2)	C22B—C21B—C20B	120.3 (2)
C22A—C21A—H21A	119.8	C22B—C21B—H21B	119.9
C20A—C21A—H21A	119.8	C20B—C21B—H21B	119.9
C23A—C22A—C21A	119.8 (2)	C21B—C22B—C23B	119.7 (2)
C23A—C22A—H22A	120.1	C21B—C22B—H22B	120.2
C21A—C22A—H22A	120.1	C23B—C22B—H22B	120.2
C22A—C23A—C24A	120.3 (2)	C22B—C23B—C24B	120.1 (2)
C22A—C23A—H23A	119.9	C22B—C23B—H23B	119.9
C24A—C23A—H23A	119.9	C24B—C23B—H23B	119.9
C19A—C24A—C23A	120.7 (2)	C19B—C24B—C23B	120.2 (2)
C19A—C24A—H24A	119.7	C19B—C24B—H24B	119.9
C23A—C24A—H24A	119.7	C23B—C24B—H24B	119.9
			
C17A—N1A—C1A—C2A	−128.16 (18)	C17B—N1B—C1B—C2B	−126.23 (17)
C9A—N1A—C1A—C2A	50.0 (2)	C9B—N1B—C1B—C2B	41.4 (2)
N1A—C1A—C2A—C7A	−45.8 (2)	N1B—C1B—C2B—C7B	−43.7 (2)
N1A—C1A—C2A—C3A	132.42 (19)	N1B—C1B—C2B—C3B	138.19 (18)
C7A—C2A—C3A—C4A	0.2 (3)	C7B—C2B—C3B—C4B	−0.2 (3)
C1A—C2A—C3A—C4A	−178.02 (19)	C1B—C2B—C3B—C4B	177.83 (18)
C2A—C3A—C4A—C5A	0.3 (3)	C2B—C3B—C4B—C5B	1.1 (3)
C3A—C4A—C5A—C6A	−0.3 (3)	C3B—C4B—C5B—C6B	−0.6 (4)
C4A—C5A—C6A—C7A	−0.2 (3)	C4B—C5B—C6B—C7B	−0.9 (3)
C5A—C6A—C7A—C2A	0.7 (3)	C3B—C2B—C7B—C6B	−1.2 (3)
C5A—C6A—C7A—C8A	−179.92 (17)	C1B—C2B—C7B—C6B	−179.39 (17)
C3A—C2A—C7A—C6A	−0.7 (3)	C3B—C2B—C7B—C8B	177.60 (17)
C1A—C2A—C7A—C6A	177.64 (16)	C1B—C2B—C7B—C8B	−0.6 (2)
C3A—C2A—C7A—C8A	179.90 (16)	C5B—C6B—C7B—C2B	1.8 (3)
C1A—C2A—C7A—C8A	−1.8 (2)	C5B—C6B—C7B—C8B	−176.95 (18)
C6A—C7A—C8A—C9A	−131.58 (18)	C2B—C7B—C8B—C9B	48.4 (2)
C2A—C7A—C8A—C9A	47.8 (2)	C6B—C7B—C8B—C9B	−132.84 (19)
C17A—N1A—C9A—C10A	−67.8 (2)	C17B—N1B—C9B—C10B	−67.7 (2)
C1A—N1A—C9A—C10A	114.07 (17)	C1B—N1B—C9B—C10B	124.96 (16)
C17A—N1A—C9A—C8A	172.49 (16)	C17B—N1B—C9B—C8B	171.73 (15)
C1A—N1A—C9A—C8A	−5.6 (2)	C1B—N1B—C9B—C8B	4.3 (2)
C7A—C8A—C9A—N1A	−42.5 (2)	C7B—C8B—C9B—N1B	−48.7 (2)
C7A—C8A—C9A—C10A	−163.24 (15)	C7B—C8B—C9B—C10B	−170.89 (16)
C11A—N2A—C10A—O1A	−1.8 (3)	C11B—N2B—C10B—O1B	2.6 (3)
C11A—N2A—C10A—C9A	175.61 (16)	C11B—N2B—C10B—C9B	179.40 (16)
N1A—C9A—C10A—O1A	−43.4 (2)	N1B—C9B—C10B—O1B	−47.3 (2)
C8A—C9A—C10A—O1A	77.4 (2)	C8B—C9B—C10B—O1B	74.0 (2)
N1A—C9A—C10A—N2A	139.05 (16)	N1B—C9B—C10B—N2B	135.71 (16)
C8A—C9A—C10A—N2A	−100.12 (18)	C8B—C9B—C10B—N2B	−102.99 (18)
C10A—N2A—C11A—C16A	169.06 (18)	C10B—N2B—C11B—C16B	151.23 (19)
C10A—N2A—C11A—C12A	−12.0 (3)	C10B—N2B—C11B—C12B	−30.9 (3)
C16A—C11A—C12A—C13A	0.1 (3)	C16B—C11B—C12B—C13B	−1.7 (3)
N2A—C11A—C12A—C13A	−178.81 (19)	N2B—C11B—C12B—C13B	−179.6 (2)
C11A—C12A—C13A—C14A	0.3 (4)	C11B—C12B—C13B—C14B	0.8 (4)
C12A—C13A—C14A—C15A	−0.3 (4)	C12B—C13B—C14B—C15B	0.5 (4)
C13A—C14A—C15A—C16A	−0.3 (4)	C13B—C14B—C15B—C16B	−0.9 (3)
C14A—C15A—C16A—C11A	0.8 (3)	C14B—C15B—C16B—C11B	−0.1 (3)
C12A—C11A—C16A—C15A	−0.7 (3)	C12B—C11B—C16B—C15B	1.4 (3)
N2A—C11A—C16A—C15A	178.33 (19)	N2B—C11B—C16B—C15B	179.33 (19)
C9A—N1A—C17A—O2A	−179.46 (17)	C18B—O3B—C17B—O2B	1.7 (2)
C1A—N1A—C17A—O2A	−1.3 (3)	C18B—O3B—C17B—N1B	−178.90 (14)
C9A—N1A—C17A—O3A	0.5 (2)	C9B—N1B—C17B—O2B	−173.78 (17)
C1A—N1A—C17A—O3A	178.60 (15)	C1B—N1B—C17B—O2B	−6.2 (3)
C18A—O3A—C17A—O2A	−4.1 (3)	C9B—N1B—C17B—O3B	6.8 (2)
C18A—O3A—C17A—N1A	175.97 (16)	C1B—N1B—C17B—O3B	174.36 (14)
C17A—O3A—C18A—C19A	−154.89 (17)	C17B—O3B—C18B—C19B	−152.18 (16)
O3A—C18A—C19A—C24A	−126.1 (2)	O3B—C18B—C19B—C24B	−140.1 (2)
O3A—C18A—C19A—C20A	55.3 (3)	O3B—C18B—C19B—C20B	44.1 (3)
C24A—C19A—C20A—C21A	0.1 (4)	C24B—C19B—C20B—C21B	−0.8 (3)
C18A—C19A—C20A—C21A	178.8 (2)	C18B—C19B—C20B—C21B	175.0 (2)
C19A—C20A—C21A—C22A	0.1 (4)	C19B—C20B—C21B—C22B	−0.8 (4)
C20A—C21A—C22A—C23A	−0.1 (4)	C20B—C21B—C22B—C23B	1.1 (4)
C21A—C22A—C23A—C24A	−0.1 (4)	C21B—C22B—C23B—C24B	0.3 (4)
C20A—C19A—C24A—C23A	−0.3 (3)	C20B—C19B—C24B—C23B	2.2 (4)
C18A—C19A—C24A—C23A	−179.0 (2)	C18B—C19B—C24B—C23B	−173.7 (2)
C22A—C23A—C24A—C19A	0.3 (4)	C22B—C23B—C24B—C19B	−1.9 (4)

### Hydrogen-bond geometry (Å, º)

**Table:**

*D*—H···*A*	*D*—H	H···*A*	*D*···*A*	*D*—H···*A*
N2*A*—H2*A*···O1*B*^i^	0.97 (1)	1.97 (1)	2.9213 (16)	167 (1)
N2*B*—H2*B*···O2*B*^ii^	0.97 (1)	1.92 (1)	2.8904 (16)	176 (1)

Symmetry codes: (i) −*x*+1, *y*−1/2, −*z*; (ii) −*x*+2, *y*−1/2, −*z*.

## References

[bb1] Dolomanov, O. V., Bourhis, L. J., Gildea, R. J., Howard, J. A. K. & Puschmann, H. (2009). *J. Appl. Cryst.* **42**, 339–341.

[bb3] Naicker, T., Govender, T., Kruger, H. G. & Maguire, G. E. M. (2011*a*). *Acta Cryst.* E**67**, o67. 10.1107/S1600536810050361 PMC3050362 21522778

[bb4] Naicker, T., Govender, T., Kruger, H. G. & Maguire, G. E. M. (2011*b*). *Acta Cryst.* E**67**, o1106. 10.1107/S1600536811012554 PMC3089297 21754424

[bb5] Nonius (2000). *COLLECT* Nonius BV, Delft, The Netherlands.

[bb6] Otwinowski, Z. & Minor, W. (1997). *Methods in Enzymology*, Vol. 276, *Macromolecular Crystallography*, Part A, edited by C. W. Carter Jr & R. M. Sweet, pp. 307–326. New York: Academic Press.

[bb7] Peters, B. K., Chakka, S. K., Naicker, T., Maguire, G. E. M., Kruger, H. G., Andersson, P. G. & Govender, T. (2010). *Tetrahedron Asymmetry*, **21**, 679–687.

[bb8] Sheldrick, G. M. (2008). *Acta Cryst.* A**64**, 112–122. 10.1107/S0108767307043930 18156677

[bb9] Sridharan, V., Suryavanshi, P. A. & Menendez, J. C. (2011). *Chem. Rev.* **111**, 7157–7259. 10.1021/cr100307m 21830756

